# Fostering quality of life in young adults living with multiple sclerosis: a pilot study of a co-created integrated intervention

**DOI:** 10.3389/fpsyg.2024.1342166

**Published:** 2024-03-26

**Authors:** Silvia Poli, Valeria Donisi, Maria Angela Mazzi, Francesca Gobbin, Giorgia Giusto, Riccardo Orlandi, Federico Schena, Lidia Del Piccolo, Roshan das Nair, Alberto Gajofatto, Michela Rimondini

**Affiliations:** ^1^Section of Clinical Psychology, Department of Neuroscience, Biomedicine and Movement Science, University of Verona, Verona, Italy; ^2^Section of Neurology, Department of Neuroscience, Biomedicine and Movement Science, University of Verona, Verona, Italy; ^3^Section of Movement Science, Department of Neuroscience, Biomedicine and Movement Science, University of Verona, Verona, Italy; ^4^Department of Health Research, SINTEF Digital, Trondheim, Norway; ^5^School of Medicine, University of Nottingham, Nottingham, United Kingdom

**Keywords:** multiple sclerosis, quality of life, wellbeing, biopsychosocial approach, feasibility, contextual-behavioral therapies

## Abstract

**Introduction:**

Multiple sclerosis (MS) is generally diagnosed at an early age, making the acceptance of this chronic disease challenging. Research dedicated to young adults with MS (YawMS) is still limited. A biopsychosocial co-created intervention for YawMS integrating social, physical and psychological activities was developed (ESPRIMO intervention) in order to improve the quality of life (QoL) and well-being. This pre-post intervention assessment study examines the feasibility of the ESPRIMO intervention and its signal of efficacy.

**Methods:**

Inclusion criteria were: age 18–45 years, MS diagnosis, Expanded Disability Status Scale score < 3.5. After giving informed consent, YawMS completed a battery of questionnaires, which was repeated after the intervention. The battery included a bespoke feasibility scale, the COOP/WONCA charts, and the Short Form-12 Health Survey (SF-12).

**Results:**

Fifty-three YAwMS were enrolled and 43 (81.1%) completed the intervention. The majority of the sample positively rated the pleasantness, usefulness and feasibility of the intervention. A significant change in the COOP/WONCA “general QoL” chart (*t* = 3.65; *p* < 0.01) and SF-12 mental wellbeing component (*t* = −3.17; *p* < 0.01) was found.

**Discussion:**

ESPRIMO is an innovative intervention that is feasible; preliminary results show an improvement in QoL and mental wellbeing. Further studies are needed to test its efficacy and evaluate future implementation in health services.

**Clinical trial registration**: ClinicalTrials.gov, NCT04431323.

## Introduction

Multiple Sclerosis (MS) is a chronic inflammatory disease of the central nervous system characterized by the presence of areas of demyelination and axonal loss in the brain and spinal cord (Filippi et al., 2019). MS is a multifactorial, immune-mediated disease; the immune system attacks the nervous system causing lesions, called plaques (Filippi et al., 2018; Li et al., 2018). Depending on the location and extent of the plaques and other pathological mechanisms, a wide spectrum of neurological symptoms may occur, such as impaired motor function, visual problems, sensory disturbances, and cognitive deficits. Italy is considered a high-risk area for MS (Battaglia and Bezzini, 2017). According to recent estimates by the Italian Society of Multiple Sclerosis, there is a prevalence of about 122,000 cases and an incidence of 3,400 cases per year.

Multiple Sclerosis has a significant impact on young adults; in fact, it is considered the most common chronic neurological disease causing disability in young adults. Moreover, MS is generally diagnosed in early age, between the ages of 20 and 40, a stage of life of great significance for the achievement of personal, professional, and relational goals. The characteristics of the disease, such as complexity and unpredictability of the clinical course, lead people to face uncertainty and the lack of control over health (Dennison et al., 2009; Wilkinson and Nair, 2013). Therefore, receiving a diagnosis of MS during early adulthood makes acceptance of this chronic disease particularly challenging (Pagnini et al., 2014; Strober, 2018; Calandri et al., 2019; Gajofatto et al., 2019), and a recent review has highlighted the need for studies on interventions to support adjustment following MS diagnosis (Topcu et al., 2020).

The process of adapting to the disease involves psychological, social, as well as physical factors (Gajofatto et al., 2019); considering the clinical course of MS, patients need to continuously find new ways to adapt. During this journey, people with MS might experience psychological distress and the literature underlines a higher risk of developing depression and anxiety compared to the general population (Boeschoten et al., 2017). Symptoms of depression have implications for the physical health and psychosocial wellbeing of people with MS, leading to worsening of the disease, greater isolation, decreased adherence to medications and increased pain (Kidd et al., 2017). Moreover, people with MS tend to report reduced health-related quality of life (HRQoL; Grossman et al., 2010; Pappalardo et al., 2017; Rainone et al., 2017; Gil-González et al., 2021), which is a multidimensional concept that focuses on the impact of the disease and/or treatments on the person’s perception of their physical, emotional and social functioning, of their health status and of their satisfaction (Megari, 2013; Pappalardo et al., 2017).

Considering the impact of MS, providing young people with effective strategies to maintain quality of life and wellbeing after the diagnosis is a key priority not only to prevent emotional distress but also to foster adjustment to the disease and its clinical course. Enhancing protective factors and resilient adaptation may contribute to a higher HRQoL of people with MS (Kasser and Zia, 2020) allowing YawMS to develop their talents and flourish despite the disease.

Research dedicated to YawMS is still scarce, as intervention, typically, do not target specific age groups. For instance, a review by Fiest et al. (2016), shows that the inclusion criteria for age in interventions for people with MS are broad (e.g., targeting people over 18 years old or people between 18 and 75 years old). Therefore, better knowledge on how to promote wellbeing and HRQoL in this specific population is needed (Rainone et al., 2017; Silverman et al., 2017; Calandri et al., 2019). Furthermore, a biopsychosocial approach, although necessary to understand the complex factors that influence HRQoL, is rarely considered in the design and implementation of interventions (Donisi et al., 2021b).

The ESPRIMO project started in 2018 with the aim of addressing the gaps in the literature and in healthcare services by developing a biopsychosocial co-created intervention for YawMS (Donisi et al., 2021b). The primary aim of the present study is to examine the feasibility of the ESPRIMO intervention. The hypothesis was that ESPRIMO would be feasible. The secondary aim of the study was to explore the signal of efficacy of the ESPRIMO intervention in improving HRQoL and wellbeing. We hypothesized that ESPRIMO would show a positive impact on these outcomes.

## Methods

The current study follows the latest revision of the Helsinki declaration, and the Oviedo declaration; it has been approved by the Ethical Committee of the Verona Hospital (Prog. 2676CESC) and prospectively registered (ClinicalTrials.gov; ID: NCT04431323). The study design was a pre-post intervention assessment. The age range of 18–45 years was used in this study to define “young adult,” as there is no clear age cutoff in the literature. This range is consistent with previous research in the medical field (Garcia and Finlayson, 2005; Pezzini, 2012; Andersson and Vasan, 2018), including MS patients (Jensen et al., 2012), and represents a slightly widened age range of MS disease onset (i.e., 20–40 years).

### Participants’ recruitment and study procedures

The ESPRIMO intervention targeted YawMS according to the following inclusion criteria: age range 18–45 years, MS diagnosis as reported by the treating neurologist in the medical record according to the revised McDonald Criteria (Thompson et al., 2018), Italian speakers, and signed informed consent. As an additional inclusion criterion, the baseline assessment phase and the subsequent intervention could be started if patients were stable on disease-modifying therapies for at least 1 month and if there had not been relapses in the last 3 months. For further details, see the protocol description (Donisi et al., 2021b).

The exclusion criteria included: severe psychiatric disorders (as evaluated by the treating neurologist or the clinical psychologist on the basis of the medical record), clinically relevant cognitive deficits (as evaluated by the treating neurologist); and clinically relevant physical impairments defined as an Expanded Disability Status Scale (EDSS) score higher than 3.5 (Bowen et al., 2001). Recruitment was conducted by neurologists/residents working at the MS Center of Verona University Hospital (MS Hub Center, northeast of Italy) and affiliated spoke clinics. During the patient’s visit, the neurologists/residents explained the study and assessed inclusion/exclusion criteria. The clinical psychologists, working at the Clinical Psychology Unit of the Verona University Hospital, contacted people willing to participate to allocate them to the available intervention group session.

After giving informed consent, all participants completed a battery of self-report questionnaires pre-treatment (T0—within 1 week before treatment), post-treatment (T1—within 1 week after treatment), and at follow-up (T2—within 1 month after the end of the intervention after a booster session; only a brief version of the battery was administered). Treatment feasibility was evaluated at the end of the intervention (T1). For the purposes of the current paper, the focus will be on the treatment feasibility and the signals of efficacy on the main outcomes, namely HRQoL and wellbeing (see Donisi et al., 2021b for the list of all evaluated variables).

According to the study protocol (Donisi et al., 2021b), participants were considered as dropouts if their attendance to the intervention was lower than 75%. However, considering that during the piloting phase, COVID-19 control measures were enforced and, in the event of close contact with a COVID-19 positive person, people had to isolate themselves at home and thereby miss sessions, a higher rate of nonattendance was set. Therefore, YaWMS were considered dropouts if they missed six (50%) or more encounters; treatment completers and dropouts were compared on sociodemographic and clinical characteristics.

### Measures

#### Socio-demographic and clinical characteristics

Sociodemographic and clinical characteristics (e.g., age, gender, marital status, educational level, employment, living situation, MS type, and months since diagnosis) were collected at T0 using routine clinical and administrative forms and a bespoke questionnaire.

#### Treatment feasibility

At T1, a questionnaire developed by the authors was administered to evaluate the acceptance and satisfaction of the intervention on utility (if the intervention was useful according to participants), feasibility (if the intervention was practical), and pleasantness (if the intervention was enjoyable). The participant self-reported questions regarding feasibility were rated on a Likert scale ranging from 1 (not at all) to 10 (very much), with higher scores reflecting higher levels of utility, feasibility, and pleasantness.

#### Health-related quality of life

The COOP/WONCA charts (van Weel et al., 1995; Pappalardo et al., 2017) was used to measure HRQoL. The COOP/WONCA comprise six charts, each representing a different aspect of functional status: physical fitness, feelings, daily activities, social activities, change in health, and overall health.

Each chart consists of a title, a question referring to the status of the person during the past 2 weeks and, as possible responses, five options accompanied by a depiction. Each chart is rated on a five-point Likert scale ranging from 1 (no limitation at all) to 5 (severely limited); for “change in health” a score of 1 means “much better” and a score of 5 “much worse.” For the current project, the chart regarding feeling was expanded, adding a chart for each feeling listed in the original question (anxious, depressed, irritable, downhearted, and sad).

“Overall health” was used as the primary measure for the current study (for detail about the power analysis see the protocol description; Donisi et al., 2021b). The COOP/WONCA charts were administered to participants at T0, T1, and T2 to assess the stability of the results over time.

The charts have proven to be reliable and valid in different medical conditions and have already been used with an Italian cohort of people with MS (Pappalardo et al., 2017). In our sample, the internal consistency for the summary score was 0.81 (range: 0.77–0.84), as measured by Cronbach’s alpha on baseline data.

As an additional measure of quality of life, two items of the Multiple Sclerosis Quality of Life-54 (MSQoL-54) questionnaire (Vickrey et al., 1995) were administered. The MSQOL-54 is an HRQoL measure that comprises 54 items organized into 12 subscales. In our study, the two items related to the overall quality of life scale were included, which tap global subjective assessment of the overall quality of life. One item is a quality of life criterion measure and the other item asks respondents to assess their satisfaction with their life in general, using a seven-point “delighted-terrible” scale.

#### Wellbeing

The 12-Item Short-Form Health Survey (SF-12) (Ware et al., 1996; Apolone et al., 2005) allows people to describe the state of health through two synthetic indices (physical and mental component summary, PCS and MCS, respectively) calculated starting from the 12 questions addressed to the respondent. The 12 questions refer to the past month; a higher score indicates a higher perception of wellbeing. The PCS has six questions covering physical functioning, role limitations due to physical health problems, bodily pain, and general health. The MCS has six questions covering mental health, role limitations due to personal problems or emotional distress, social functioning, and vitality.

PCS and MCS are expressed in standardized T scores ranging from 0 to 100. These scores were obtained using specific algorithms; we used the algorithms from a study that adapted the instrument in Italy (Ottoboni et al., 2017). SF-12 has been used in studies with people with MS (Gasper et al., 2020), and in the Italian context (Calandri et al., 2017). In our sample, the estimated internal consistency for PCS score was 0.87 (range: 0.82–0.86) and 0.87 (range: 0.82–0.87) for MCS.

### Treatment description

**Figure 1:**
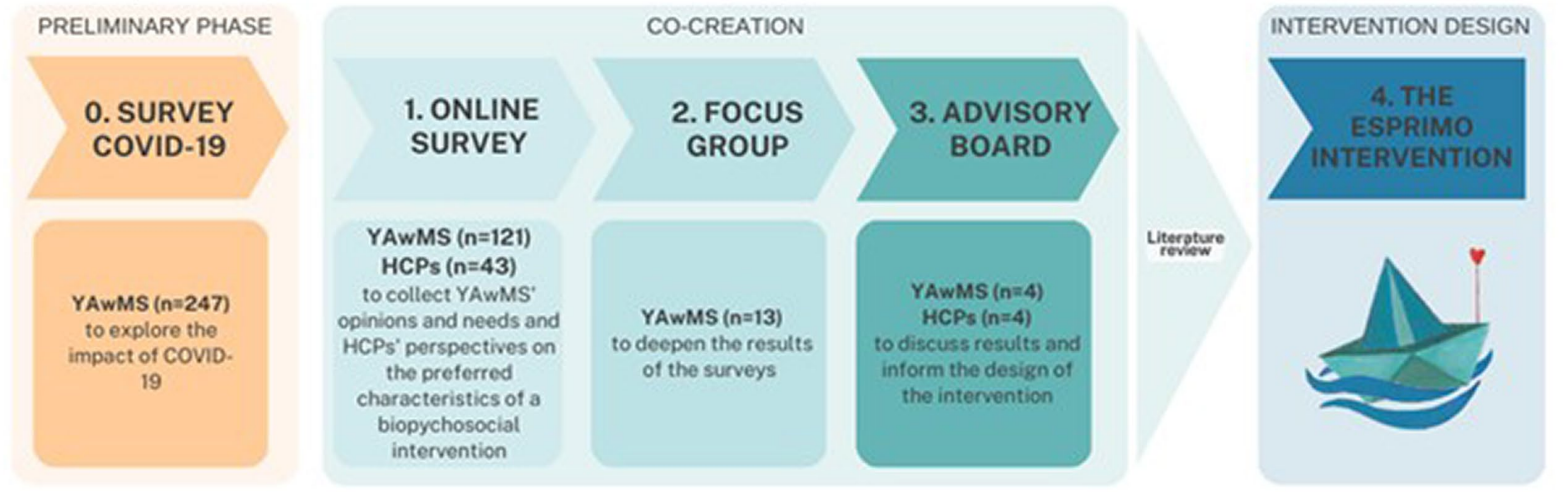
Overview of the co-creation process. The ESPRIMO study was conducted during the COVID-19 pandemic; thus, in a preliminary phase, a survey was conducted after the end of the lockdown in Italy (May 2020) (Donisi et al., 2021a; Poli et al., 2021).

The ESPRIMO intervention was designed using a co-creation approach (Morote et al., 2020; Donisi et al., 2022) that actively involved the main stakeholders in the management of MS. Researchers and healthcare professionals with different backgrounds (i.e., neurologists, psychologists, nurses, and sport scientists) and young adults with MS were consulted through all three steps of the co-creation (i.e., surveys, focus groups, consultation with the Advisory Board) aimed at collecting the preferences and opinions of people with MS and healthcare professionals to outline the objectives, methodology, and practical implementation of the intervention, integrating the results with the evidence available in the literature. Figure 1 describes the co-creation methods (Donisi et al., 2022).

**Figure 2:**
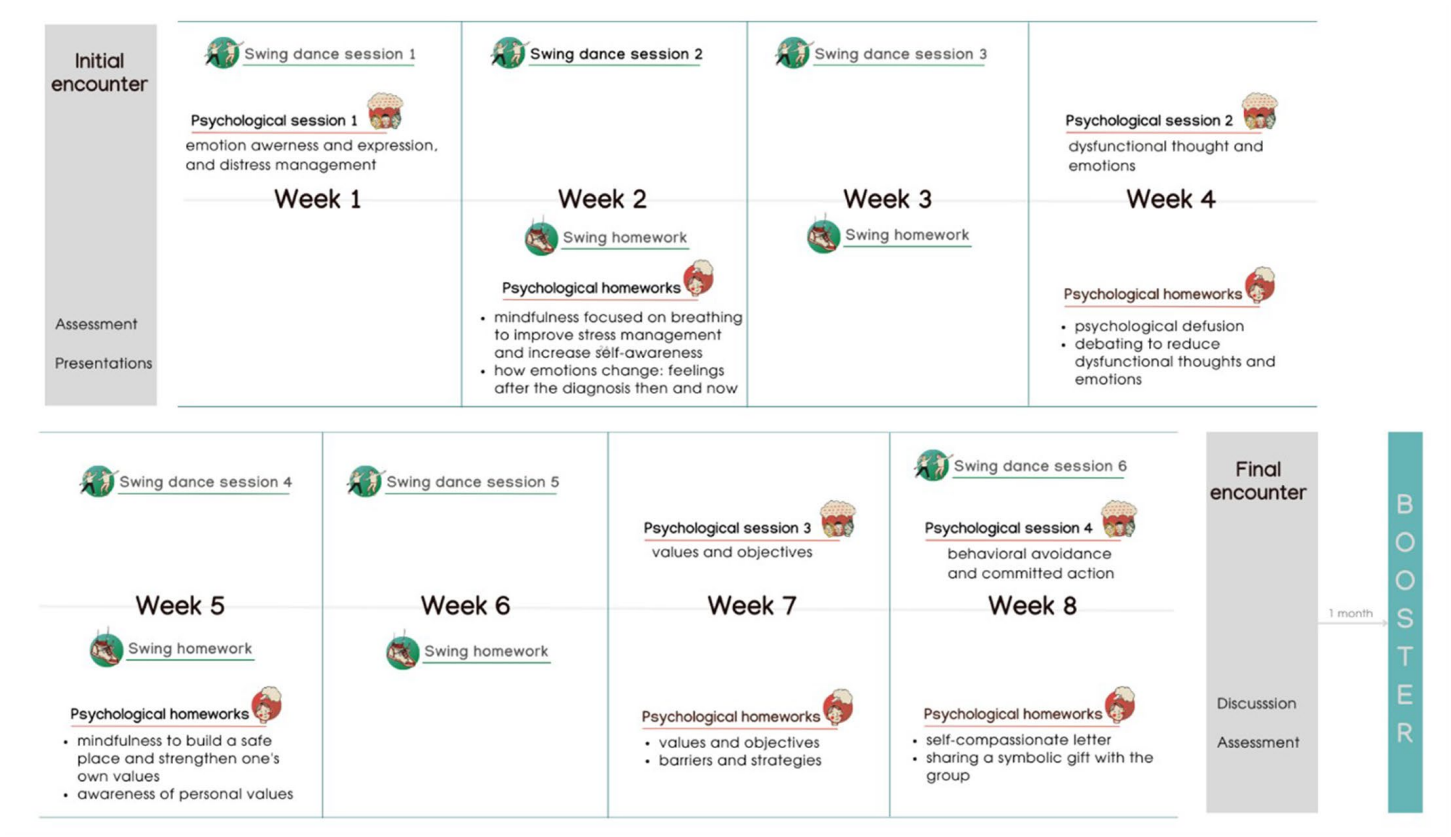
Overview of the ESPRIMO intervention program.

ESPRIMO is a biopsychosocial intervention that integrates physical activity, psychological support, and social interaction. It lasts 10 weeks with 12 total in-person group encounters, plus one booster session 1 month after the end of the intervention (see Figure 2). In addition to the group activities, participants were invited to complete short psychological and physical exercises that were guided by an illustrated manual and by videos or audios that were shared weekly on a dedicated ESPRIMO Telegram channel.

The swing course/activities had the aim to teach participants a dance routine: in each session participants learned new steps and rehearsed the other steps. To exercise, participants were sent on the Telegram channel videos of the steps performed by the instructors. The manual offered some space to reflect on the progress and, for example, on emotions experienced during the in-group physical activities.

Psychological group session were guided by cognitive behavioral therapy, mindfulness, positive psychology, and acceptance and commitment therapy as main theoretical approaches (Donisi et al., 2021b). Based on the co-creation process, the main aims were to help people accept the disease, provide stress management strategies, support emotional expression and regulation, and increase self-efficacy in managing the disease (Donisi et al., 2022).

The manual and the exercises were designed to help participants review the main concepts, foster reflection and learning, and provide additional psychological techniques (e.g., mindfulness exercise to improve stress management and increase self-awareness; debating exercise to reduce dysfunctional thoughts).

Regarding the social component, the use of small groups was chosen to allow the sharing of experiences between participants, to encourage peer support, and to foster sense of belonging. The Telegram channel also allowed for informal communication between participants and acted as a space to share thoughts, doubts, or improvements. The social aspect was further promoted through inter-group events. One month after the end of the intervention, a booster event was held that included an informal event (with dance activities) open to the participants, their families and the whole community.

Table 1 provides a description of the ESPRIMO intervention in accordance with the TIDieR checklist for better reporting of interventions (Hoffmann et al., 2014).

**Table 1:** Description of the ESPRIMO intervention based on the TiDIER checklist (Hoffmann et al., 2014).

*N*°	Item	
1	Lay title	Biopsychosocial co-created intervention to promote quality of life in young adults with multiple sclerosis
2	Why	An integrated biopsychosocial intervention for people with MS can enhance HRQoL and wellbeing. The rationale for the intervention contents and structure was based on a participatory approach (Donisi et al., 2021b, 2022)
3	What (materials)	Intervention manual for participants including homework and sessions summaries. Telegram Channel to share audios and videos. Presentation slides and notes to guide intervention delivery and enhance fidelity.
4	What (procedure)	The ESPRIMO intervention integrates physical activity, psychological support, and social interaction by offering dance and psychological sessions delivered in small groups of YawMS. In addition to group sessions, the intervention includes individual homework and a telegram channel. The group sessions included: An initial encounter: after the pre-intervention assessment, the intervention program was discussed, and participants and the ESPRIMO team presented themselves. Swing dance sessions (six sessions): after a warming-up exercise, few steps were taught (e.g., rock step, triple step) with a progression in the difficulty as weeks went by, with the aim to teach participants a dance routine. Psychological sessions (four sessions): after a warm-up, the clinical psychologist briefly explained the topic of the session and then facilitated the discussion allowing the sharing of experiences of participants. The sessions focused on: (1) emotion awareness, expression, and distress management, (2) dysfunctional thoughts and emotions, (3) values and objectives, and (4) behavioral avoidance and committed action. A final encounter: discussion with reflection and future objectives; after the discussion participants were asked to complete the post-treatment assessment. A booster session (1 month after the end of the intervention): a 40-min follow-up psychological session aiming to consolidate the psychological strategies followed by an informal event that welcomed all ESPRIMO participants (including the ones of the previous groups) and their family/friends. During the informal event, the swing routine was practiced by the participants and all the persons that wished to join.
5	Who provided (facilitator)	The swing sessions were led by two swing-trained specialists. Psychological sessions were facilitated by one licensed clinical psychologist with training in cognitive behavioral therapy and acceptance and commitment therapy, and with clinical experience in the MS field. A clinical phycologist was always present during both psychological and dance sessions (to serve as a reference person during the intervention) and managed the delivery of homework.
6	How (Method of delivery)	Sessions were held in-person weekly (one each week except for 2 weeks that had two encounters each) with a total duration of 10 weeks. Groups had an average of eight participants. Participants could complete homework during the week using the manual and the guided videos/audios available on the Telegram channel.
7	Where	Participants were recruited at the MS Center of Verona University Hospital and the affiliated SPOKE clinics. All group activities were held in a neutral, easily accessible venue surrounded.
8	When and how much	The ESPRIMO intervention had 12 in-person group sessions: one initial encounter, six swing dance motor sessions (lasting 1 h) and four psychological sessions (lasting 1.5 h), one final encounter, and one booster session. Homework regarding physical and psychological activities was administered weekly.
9	Tailoring	Different timeframes for the group sessions (lunch break, afternoon, and evening) in order to foster participation. Psychologists had some flexibility in the explanation of the topic based on the participants’ feedbacks (e.g., using specific examples relevant to the group). The swing dance specialists could adapt the activities based on the participants’ physical abilities. Alternative steps were available if needed depending on the participants’ level of ability.
10	Modification	The intervention was delivered during the COVID-19 pandemic. Criteria for quarantine, and the use of personal protective equipment had to be adapted to follow the restrictions enforced by the Government.

### Data analysis

Descriptive statistics were presented as mean values and standard deviation (SD) for continuous variables and as frequencies for categorical variables. Chi-squared, Fisher exact tests, and independent *t*-test were used to explore the differences in sociodemographic and clinical characteristics between treatment completers and dropouts. Student’s paired *t*-test was run to explore changes in HRQoL and wellbeing, at the level of post-treatment and follow-up, using pre-treatment values as reference. Effect size estimation was calculated using Cohen’s dp, based on the pooled sample standard deviation (Goulet-Pelletier and Cousineau, 2018), which standardizes the magnitude of effect and facilitates the comparison between studies; its 95% confidence interval was estimated by using the Algina-Keselman method (Algina and Keselman, 2003). Pearson correlations were used to explore the associations among COOP/WONCA charts at different time points.

Statistical analyses were conducted with Stata and Cohensdp package, within RStudio (Cousineau, 2022).

## Results

### Recruitment and retention rates

**Figure 3:**
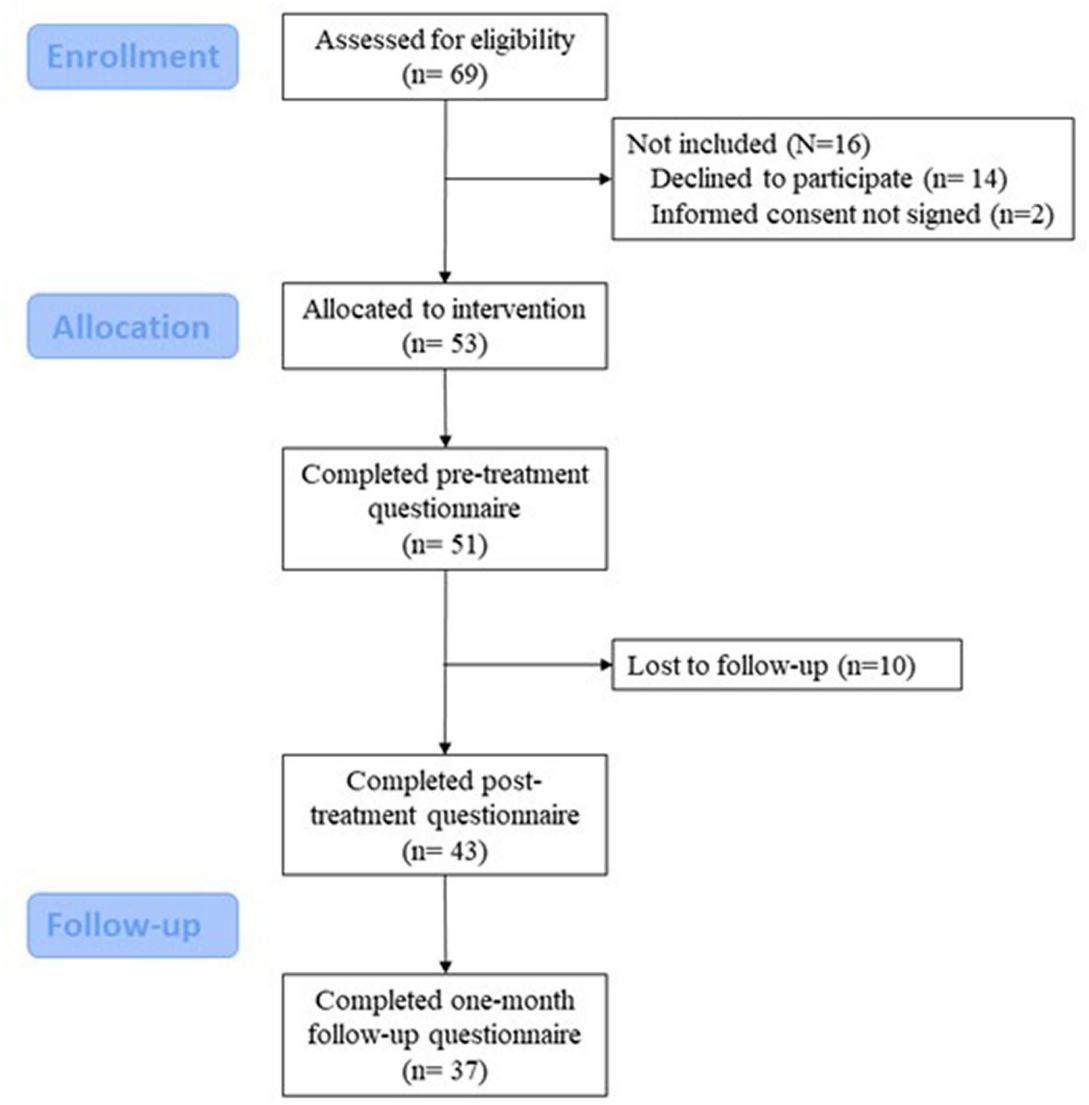
CONSORT diagram showing participants’ flow through the study.

Six rounds of the ESPRIMO intervention were run from September 2020 to July 2021; a total of 53 YAwMS were enrolled and 43 (81.1%) completed the treatment. See Figure 3 for the CONSORT diagram of the participants’ journey through the study.

Out of the 10 people who dropped out, two left after the first session, three attended only two sessions, two missed seven encounters, while the three remaining participants missed eight, nine, and six encounters. Two of the people who dropped out did not complete pre-treatment questionnaire.

### Sample characteristics

Participants had a mean age of 33.4 ± 6.8 years (age range 22–45). The majority were women (80%) and were married or living with a partner (66%) and had an academic degree (49%). Relapsing–remitting MS was the most frequent diagnosis in the sample (96%); only two participants had a diagnosis of primary progressive MS. The mean time from diagnosis was 6.3 ± 5.8 years. Mean EDSS score was 1.45 ± 1.12. Two participants were not receiving MS drugs, while the others were taking the following disease-modifying treatments: Natalizumab (*n* = 11), Interferon (*n* = 9), Glatiramer acetate (*n* = 5), Dimethylfumarate (*n* = 5), Anti-CD20 drugs (*n* = 5), Teriflunomide (*n* = 4), Cladribine (*n* = 4), Fingolimod (*n* = 3), Azathioprine (*n* = 1), Methotrexate (*n* = 1), and Alemtuzumab (*n* = 1).

The socio-demographic and clinical information, broken down by treatment completers and treatment dropouts, is shown in Table 2. Between treatment completers and dropouts, no statistically significant differences were found for age, occupation, living situation, and time since diagnosis.

**Table 2:** Socio-demographic and clinical characteristics of treatment completers and dropouts.

	Treatment completers	Dropouts	
	*n* = 43	*n* = 10	*Χ*^2^
*Gender*			
Women	37	3	13.77
Men	6	7	*p* < 0.01^*^
*Educational status*			
Secondary/professional school degree	8	3	5.36
High school degree	10	4	
Graduate degree	24	1	*p* = 0.03^*^
*Occupation*			
Yes	31	4	3.73
No	12	6	*p* = 0.06
*Living situation*			
Living with a partner/children	30	3	4.83
Living with parents	7	4	
Living with non-family cohabitants	1	0	*p* = 0.14
Alone	5	1	
Age: mean ± SD (range) years	33.9 ± 6.4 (22–45)	31.3 ± 8.2 (22–44)	*t* = 1.09; *p* = 0.28
Time since diagnosis: mean ± SD (range) years	6.3 ± 5.6 (0–26)	6.4 ± 7.3 (0–22)	*t* = 0.06; *p* = 0.96

### Treatment feasibility

Treatment completers evaluated the pleasantness, the utility and the feasibility of the intervention with a mean score of 9.1 ± 0.97, 8.7 ± 1.3, and 8.6 ± 1.4, respectively. The utility of the manual was evaluated with a mean score of 7.3 ± 2.

**Figure 4:**
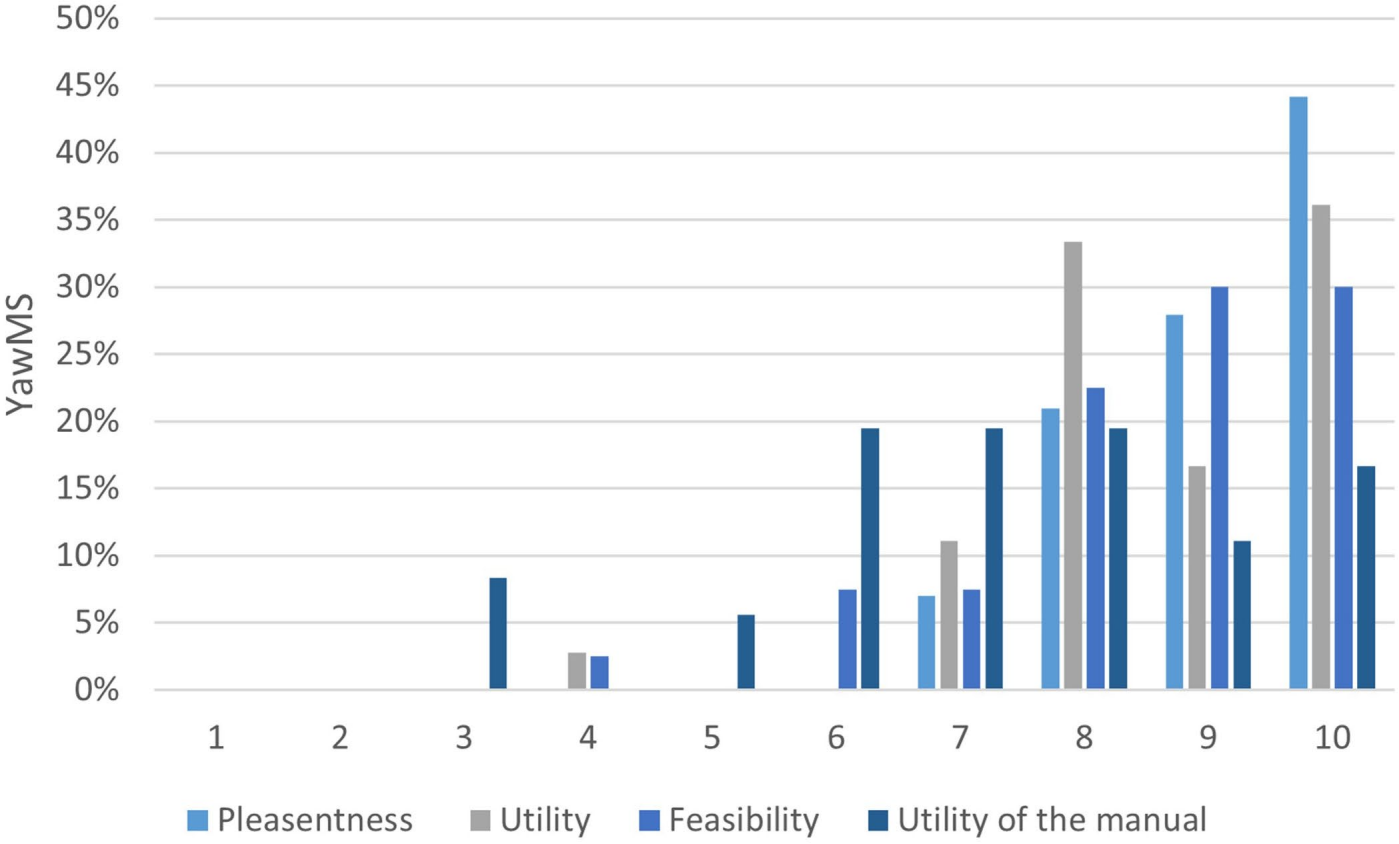
Feasibility scores of the ESPRIMO intervention program.

Figure 4 shows the rating distribution for treatment feasibility.

Among the individuals, 100, 97, and 90% rated as 7 or above the pleasantness, utility, and feasibility of the intervention, respectively. Regarding the manual, 67% rated its utility as 7 or above.

### Quality of life and wellbeing

The four indexes, measuring perceived wellbeing and HRQoL, are summarized in Table 3, as gathered at the three time points: pre-treatment (T0), post-treatment (T1), and at follow-up (T2 -after the booster session). Paired *t*-test highlighted a significant decrease in the main outcome (CW10—overall health) both at T1 and T2, corresponding to an improvement in perceived HRQoL. This was confirmed by the overall quality of life scale of MSQoL-54, with an average increase of about five points between T0 and T1; the same for the mental component scale of SF12 with a difference of about five points.

**Table 3:** Means, standard deviation, and effect sizes for the HRQoL and wellbeing outcomes (*n* = 43).

Wellbeing indexes	T0	T1	Paired test	T2	Paired test
	Mean (SD)	Mean (SD)	*t* value (*p* value)	Mean (SD)	*t* value (*p* value)
CW10—Overall health	2.93 (1.0)	2.29 (0.9)	−3.65 (*p* < 0.01)	2.13 (0.9)	−2.93 (*p* < 0.01)
SF12—Mental scale	41.04 (11.3)	46.17 (9.5)	3.17 (*p* < 0.01)	-	
SF12—Physical scale	46.12 (11.6)	43.94 (12.0)	−1.89 (0.07)	-	
MSqol54—Overall QoL	64.40 (16.4)	69.50 (13.6)	3.10 (*p* < 0.01)	71.23 (15.2)	1.70 (*p* = 0.10)

**Figure 5:**
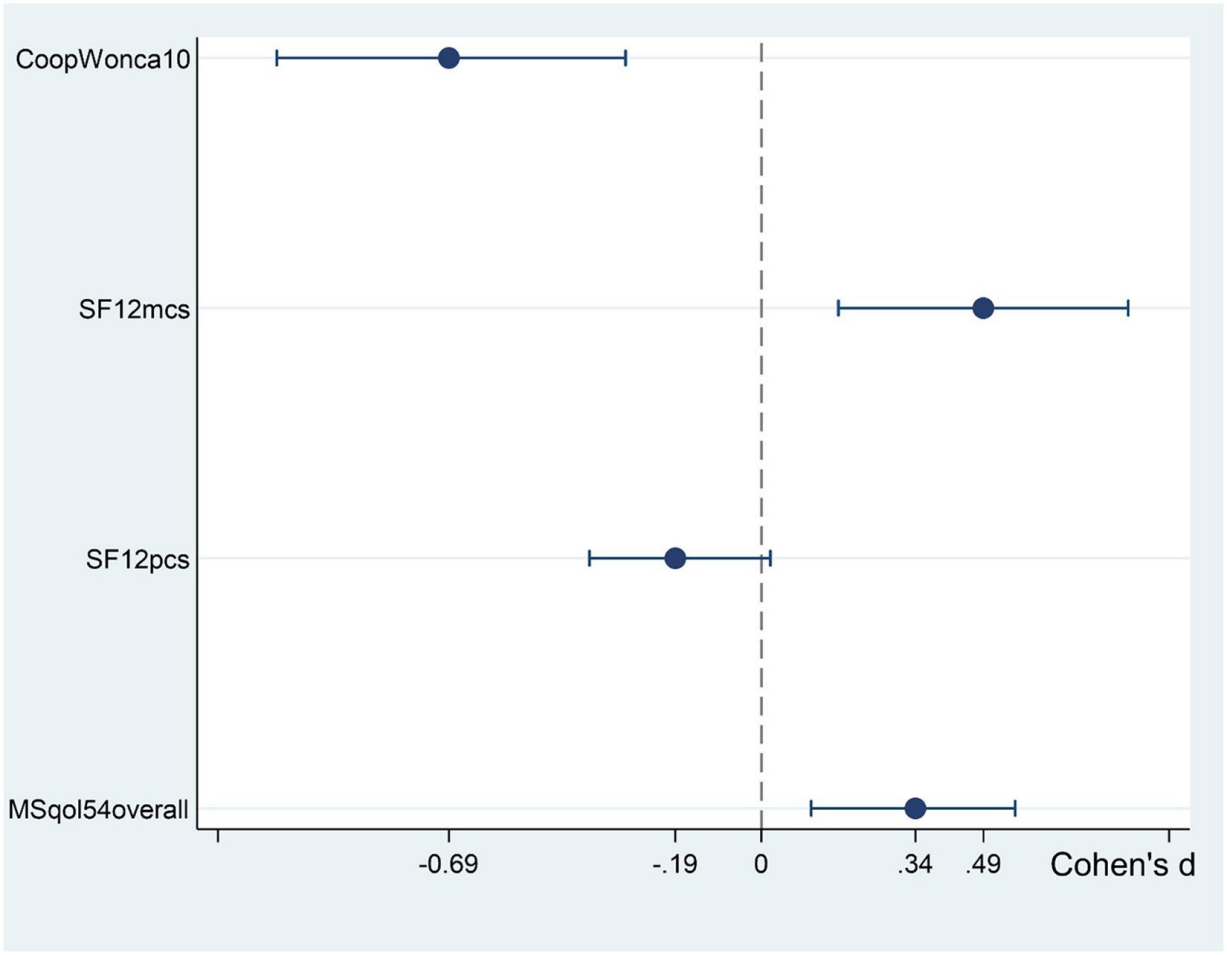
Standardized mean difference (Cohen’s effect size), with 95%CI, of wellbeing indices for the change from pre- to post-treatment. CoopWonca10, Item 10 (overall health) of the Coop/Wonca charts; SF12mcs, Mental component summary of the SF-12 questionnaire; SF12pcs, Physical component summary of the SF-12 questionnaire; and MSqol54overall, Overall quality of life scale of the MSQoL-54.

The standardized effect size is shown in Figure 5, by using Cohen’s dp formula, to compare the magnitude of the difference between pre-and post-treatment scores: the confidence intervals show statistically significant effects, except for SF12pcs. According to the literature (Lakens, 2013), the effect sized found in perceived overall health (CW10 dp = −0.69; the negative direction, following the rating score, indicates an improvement in QoL) is considered a large effect size (around 0.8), while a medium effect (around 0.5) was found in mental component scale (SF12mcs dp = 0.49), and a lower effect in overall quality of life in MS (MSqol54overall dp = 0.34).

Descriptive data of the pre- (T0) vs. post-treatment (T1) and follow-up (T2) scores of the COOP/WONCA charts are summarized in the Appendix A. Paired *t*-test highlighted a significant decrease at T1 in change in health (2.09 vs. 2.79; *t* = −4.32, *p* < 0.01) and in feelings (anxious and downhearted) at T2 (1.95 vs. 2.42, *t* = −2.28, *p* < 0.01; and 1.81 vs. 2.26; *t* = −2.32, *p* < 0.01, respectively).

After completing the ESPRIMO intervention, 56% of the participants reported a decreased score on the main outcome (CW10—overall health), while 32% of the participants had no change in score. Appendix B shows the correlation between overall health and the other COOP-WONCA charts. In the whole sample, the following charts had a relevant association with overall health: social activities, daily activities, feelings (downhearted, anxiety, sad, and depression).

## Discussion

The ESPRIMO intervention is a biopsychosocial intervention co-created with the end-users, namely, young adults with MS and absent to moderate disability (EDSS <3.5). Our findings suggest satisfactory feasibility and promising effects of the ESPRIMO intervention. Specifically, ESPRIMO was feasible, as indicated by having a satisfactory treatment completion rate of more than 80%, and high evaluations (eight or above) on pleasantness, utility, and feasibility. Moreover, promising treatment effects were supported by findings that HRQoL and mental wellbeing increased after the treatment: (i) post-treatment overall health and the overall quality of life significantly increased, (ii) more than 50% of the participants reported a decreased score on perceived overall health, and (iii) the mental wellbeing component of the SF-12 scale significantly increased at the end of the ESPRIMO intervention.

The design of the ESPRIMO intervention was informed by literature, by YawMS and healthcare professionals (Donisi et al., 2022). This co-creation process may have contributed to the feasibility and acceptability of the intervention. Starting from the preferences and needs of the end-user helps to design interventions that are more fitting and sustainable for participants, thus possibly increasing satisfaction (Lo and Karnon, 2019). In fact, in our study, the *a priori* feasibility cutoff of 6 outlined in the protocol (Donisi et al., 2021b) was fully met, with mean program satisfaction ratings being higher than 8.

Regarding the dropout rate, in randomized control trials, the acceptable threshold for the attrition rate is 20% of the randomized participants (Schulz and Grimes, 2002). Even though no threshold could be found in the literature for pre- and post-treatment comparison studies, the percentage of treatment completers in the ESPRIMO intervention was similar to other studies with people living with MS conducted in Italy (Carletto et al., 2017). Moreover, a review on self-management interventions in the MS field found that about 16% of recruited participants are expected to drop out without completing the study (Arafah et al., 2017) and a review on mindfulness-based intervention studies found that attrition ranged from 0 to 39% (Simpson et al., 2022).

The protocol and the ESPRIMO intervention (Donisi et al., 2021b, 2022) had to be adapted to address the challenges of COVID-19 and the related preventative measures enforced by the government; participants’ retention could have been influenced by these change and the pandemic itself. It has been shown that COVID-19 had an impact on clinical research with negative effects on clinical trial initiation, patient enrollment of ongoing trials, protocol adherence, clinical trials’ operations, and data collection (Lasch et al., 2022). In our study for example, one participant reported that the reason they decided to drop out was because they had missed some sessions due to COVID-19 restrictions and decided not to continue the intervention because they felt that the group had moved on in the meantime, and they felt “distant” to them.

Our results suggest that the ESPRIMO intervention had positive signals of efficacy on “overall health” after the treatment, which were maintained 1 month after the end of the intervention. Interestingly, all the charts of the COOP/WONCA (physical fitness, feelings, daily activities, social activities, and change in health) showed a trend indicating an improvement in these areas of HRQoL both post-treatment and at follow-up. These results suggest a potential effect of the intervention, which should be evaluated using a larger sample and a longer follow-up (e.g., 6 months), which might be able to capture the accumulative process of change possibly activated by the ESPRIMO intervention. Among the COOP WONCA chart, “change in health” was found to be significantly improved post-treatment and scores on the “feeling sad” and “feeling anxious” items were significantly lower 1 month after the end of the intervention. The relevance of the intervention for these dimensions is in line with the signal of effect that the intervention has for overall HRQoL and the mental wellbeing component. In fact, the mental composite score of the SF-12 had a statistically and clinically meaningful improvement (>5) suggesting positive signals of efficacy of the ESPRIMO intervention on this component of wellbeing.

The integrated approach underlying the intervention is one of the elements that might have led to the positive impact observed in our preliminary data. Mindfulness-based interventions have been effective in enhancing quality of life and in mental health in different chronic conditions (Demarzo et al., 2015; Crowe et al., 2016; Haller et al., 2017; Fisher et al., 2023) including MS (Simpson et al., 2022). Both Mindfulness-Based Stress Reduction and Acceptance and Commitment Therapy (ACT) have yielded positive results on insomnia, fatigue, paresthesia, depression emotional competencies, and stress in people living with MS (Sadeghi-Bahmani et al., 2022); however, a recent review unexpectedly did not find an improvement on quality of life after ACT (Thompson et al., 2022). The efficacy of dance as physical activity in improving quality of life has been highlighted in a previous study on people living with MS (Ng et al., 2020). Dancing seems to be beneficial for emotional wellbeing and self-esteem (Salgado et al., 2010). As for the impact of the social component, a recent review highlighted that social support and participation are positively related to quality of life and wellbeing (Gil-González et al., 2020), and a call to target social integration and social support in intervention studies to promote and maintain overall health and wellbeing has been made (Latinsky-Ortiz and Strober, 2022). These factors might have been improved by the different social activities implemented in the intervention, such as the group dimension, the Telegram channel, and the booster informal events, which might have further improved the informal network between the participants of all the ESPRIMO rounds.

It must be noted that no significant improvement in the physical composite score of the SF-12 was detected. This is surprising as dance-based interventions have been shown to improve physical outcomes such as gait, fatigue, and balance in people living with MS (Mandelbaum et al., 2016; Scheidler et al., 2018; Van Geel et al., 2020). The lack of effect on the physical composite score could be attributed to participants having had a low disability level when the intervention started, and because they were young and potentially already physically active, which was reflected in a high baseline score in the physical component. It must also be considered that two participants had a relapse of the disease during the intervention, which might have negatively impacted their perception of physical wellbeing; however, due to the small sample size, sensitivity analysis were not possible.

## Strengths and limitations

To the best of our knowledge, this is the first study that has shown that a biopsychosocial intervention is feasible and demonstrates signals of efficacy, and thus has the potential to be useful for young adults living with MS. Few MS studies have specifically focused on YawMS and psychosocial interventions for people with MS usually enroll people with an average age higher to that included in our study (Kuspinar et al., 2012; Kidd et al., 2017; Sesel et al., 2018). In the literature, one interventional study specifically targeting people older than 45 years old has been tested in the America context (Alschuler et al., 2018), while no interventional studies designed for younger adults have been tested so far. The involvement of YawMS in the design of the intervention is a strength of the ESPRIMO project. Moreover, HRQoL has been investigated using different measurement and effect sizes were calculated.

Some limitations of the study need to be acknowledged. Because feasibility was the main focus of our study, we only conducted a single-arm study with no control group. Being a feasibility study with a small sample, more sophisticated analytic strategies that could address potential confounding factors (e.g., comorbidities other than RRMS, stressful life events not related to MS, and level of physical activity) were not possible. Furthermore, even if the exact number of sessions each participant attended was recorded, analysis to predict outcomes based on the number of sessions attended were not possible. Such micro-analysis of subtle psychological changes before and after each session (Brand et al., 2018) or after every week might allow to understand more thoroughly to what extent and at which time points of the treatment improvements could be observed, thus allowing the identification of the elements of the intervention that promote change. For all these reasons, an adequately powered, randomized controlled trial with a larger sample is needed to establish whether ESPRIMO has the potential to provide positive effects. In future studies, using a longer follow-up (e.g., 6 or 12 months) might help to determine whether the intervention is acceptable and useful in the long run, detecting longitudinal trends of change after the intervention.

Most of the participants in the present study were women with relapsing–remitting MS (RRMS) and with absent or low disability. This may limit the generalizability of the results; however, the proportion of people with RRMS is consistent with the literature and with the targeted population as secondary progressive and primary progressive MS are known to begin at an older age than RRMS (Tremlett and Zhao, 2017; Filippi et al., 2018). However, even if results should be taken with caution due to the low number of male participants, the dropout rate for men was higher than for women. Moreover, people with higher level of educational status were more likely to complete the intervention. Composing more homogeneous groups in terms of gender and educational status might help to improve adherence to the intervention. Although a series of steps were taken to ensure the internal validity of our intervention, its fidelity was not formally assessed, for example by using audio/video recording. Also, we did not evaluate the costs of the ESPRIMO intervention because this fell out of the scope of the project. The group setting may have reduced costs; however, health economic evaluations should be conducted in the future to assess the sustainability of the ESPRIMO intervention.

Feasibility studies aim to answer “whether something can be done, should be done, and, if so, how” (Eldridge et al., 2016). Although future research, including a randomized control trial is needed to examine the extend and the duration of ESPRIMO efficacy, its active ingredients and costs, and possible strategies for its dissemination and implementation, ESPRIMO represents a promising and feasible approach to improve quality of life in an otherwise vulnerable population.

## Conclusion

ESPRIMO is an innovative biopsychosocial co-created intervention combining psychological, social, and physical activity. The intervention was feasible, and the preliminary results show an improvement in health-related quality of life and mental wellbeing for young adults living with MS. Such promising results in an understudied population group (i.e., young adults) and on a still not sufficiently investigated intervention approach (i.e., biopsychosocial) in the field of MS, underlines the relevance of continuing in this direction. Further evidence is needed on the impact of this integrated intervention and on its future implementation in healthcare services.

## Data availability statement

The raw data supporting the conclusions of this article will be made available by the authors, without undue reservation.

## Ethics statement

The studies involving humans were approved by Ethical Committee of the Verona Hospital (Prog 2676CESC). The studies were conducted in accordance with the local legislation and institutional requirements. The participants provided their written informed consent to participate in this study.

## Author contributions

SP: Data curation, Formal analysis, Investigation, Methodology, Project administration, Visualization, Writing – original draft, Resources. VD: Conceptualization, Data curation, Formal analysis, Investigation, Methodology, Project administration, Writing – original draft, Resources. MM: Data curation, Formal analysis, Writing – review & editing. FG: Methodology, Writing – review & editing, Resources. GG: Investigation, Resources, Writing – original draft. RO: Resources, Writing – review & editing. FS: Writing – review & editing. LP: Writing – review & editing. RN: Writing – review & editing. AG: Methodology, Writing – review & editing, Resources. MR: Conceptualization, Data curation, Formal analysis, Methodology, Project administration, Writing – original draft, Writing – review & editing, Investigation, Resources.
