# Supplementary material for: Fostering quality of life in young adults living with multiple sclerosis: a pilot study of a co-created integrated intervention
Source: Front Psychol. 2024 Mar 26;15:1342166. doi: 10.3389/fpsyg.2024.1342166 (PMC11002131; doi:10.3389/fpsyg.2024.1342166)
Supplement: Supplementary file 1 [file Table_1.DOCX]

Supplementary Material

Appendix A: Means, standard deviation and paired t-test for the COOP-WONCA charts (n=43)

| Coop Wonca  chart number and description | | Pre-treatment  mean (sd) | Post treatment  mean (sd) | t-test | Follow-up  mean (sd) | t-test |
| --- | --- | --- | --- | --- | --- | --- |
| CW1 | Physical fitness | 2.64 (0.98) | 2.54 (0.97) | -0.85 | 2.57 (1.12) | -0.36 |
| CW2 | Feelings (anxious) | 2.42 (1.10) | 2.23 (1.02) | -1.27 | 1.95 (0.88) | -2.28* |
| CW3 | Feelings (depressed) | 1.77 (0.87) | 1.62 (0.85) | -1.03 | 1.51 (0.69) | -1.29 |
| CW4 | Feelings (irritable) | 2.36 (0.91) | 2.21 (0.84) | -0.92 | 2.19 (0.91) | -0.61 |
| CW5 | Feelings (downhearted) | 2.26 (1.07) | 1.95 (0.87) | -1.91 | 1.81 (0.97) | -2.32* |
| CW6 | Feelings (sad) | 2.02 (0.99) | 2.02 (0.91) | 0.00 | 1.86 (0.82) | -0.53 |
| CW7 | Daily activities | 2.28 (1.00) | 2.05 (0.95) | -1.76 | 2.0 (0.88) | -1.56 |
| CW8 | Social activities | 1.65 (0.92) | 1.58 (0.82) | -0.42 | 1.35 (0.59) | -1.60 |
| CW9 | Change in health | 2.79 (0.86) | 2.09 (0.68) | -4.32** | 2.51 (0.80) | -0.10 |
| **CW10** | **Overall health** | **2.93 (0.96)** | **2.29 (0.90)** | **-3.65**** | **2.13 (0.90)** | **-3.49**** |
| Significant paired t-test (vs pre-treament): * p<0.05, ** p<0.01 | | | | | | |
