# Supplementary material for: Fostering quality of life in young adults living with multiple sclerosis: a pilot study of a co-created integrated intervention
Source: Front Psychol. 2024 Mar 26;15:1342166. doi: 10.3389/fpsyg.2024.1342166 (PMC11002131; doi:10.3389/fpsyg.2024.1342166)
Supplement: Supplementary file 2 [file Table_2.DOCX]

Supplementary Material

Appendix B: Correlations among COOP-WONCA (CW) charts: Overall health chart with other charts. The column “Whole sample” refers to the sum of the participants’ score in the three time points

|  | Overall health  (CW10) | PRE  (n=50) | POST  (n=41) | BOOSTER  (n=36) | Whole sample  (n=127) |
| --- | --- | --- | --- | --- | --- |
| CW1 | Physical fitness | 0.26 | 0.11 | 0.24 | 0.22 |
| CW2 | Feelings (anxious) | 0.38 | 0.40 | 0.26 | 0.37 |
| CW3 | Feelings (depressed) | 0.34 | 0.30 | 0.56 | 0.39 |
| CW4 | Feelings (irritable) | 0.10 | 0.25 | 0.19 | 0.20 |
| CW5 | Feelings (downhearted) | 0.41 | 0.42 | 0.37 | 0.44 |
| CW6 | Feelings (sad) | 0.60 | 0.46 | 0.29 | 0.46 |
| CW7 | Daily activities | 0.60 | 0.29 | 0.43 | 0.47 |
| CW8 | Social activities | 0.38 | 0.26 | 0.44 | 0.37 |
| CW9 | Change in health | 0.13 | -0.01 | 0.29 | 0.21 |
